# Targeting SREBP1 chemosensitizes colorectal cancer cells to gemcitabine by caspase-7 upregulation

**DOI:** 10.1080/21655979.2019.1676485

**Published:** 2019-10-10

**Authors:** Wenlong Shen, Ting Xu, Dong Chen, Xiaojie Tan

**Affiliations:** a Department of Anorectal, Qilu Hospital of Shandong University, Qingdao, Shandong, PR China; b Department of Geratology, The 971th Hospital of PLA, Qingdao, Shandong, China; c Department of General Surgery, The Affiliated Hospital of Qingdao University, Qingdao, Shandong, China

**Keywords:** colorectal cancer (CRC), chemotherapy, SREBP1, caspase-7, apoptosis

## Abstract

Biomarkers for predicting chemotherapy response are important for treatment of colorectal cancer (CRC) patients.SREBP1is involved in cancer cell chemoresistance, but the biological consequences of this activity in CRC are poorly understood. We set up biochemical and cell biology analyzes to analyze SREBP1 expression and chemoresistance. We found that SREBP1 was overexpressed in chemoresistant CRC samples, and that SREBP1 overexpression was correlated with poorer patient survival. Targeting SREBP1 increased chemosensitivity to gemcitabine (Gem) in CRC cells. Additionally, SREBP1 overexpression increased chemoresistance to Gem in CRC cells. SREBP1 overexpression downregulated caspase-7 and decreased CRC cell sensitivity to Gem. Low SREBP1 expression was correlated with high caspase-7 expression in CRC patient samples. Low caspase-7 expression was correlated with poor patient survival. Our findings indicated that upregulation of caspase-7 caused by downregulation of SREBP1 may be a novel prognostic biomarker, and may represent a new therapeutic target in CRC.

## Graphical Abstract

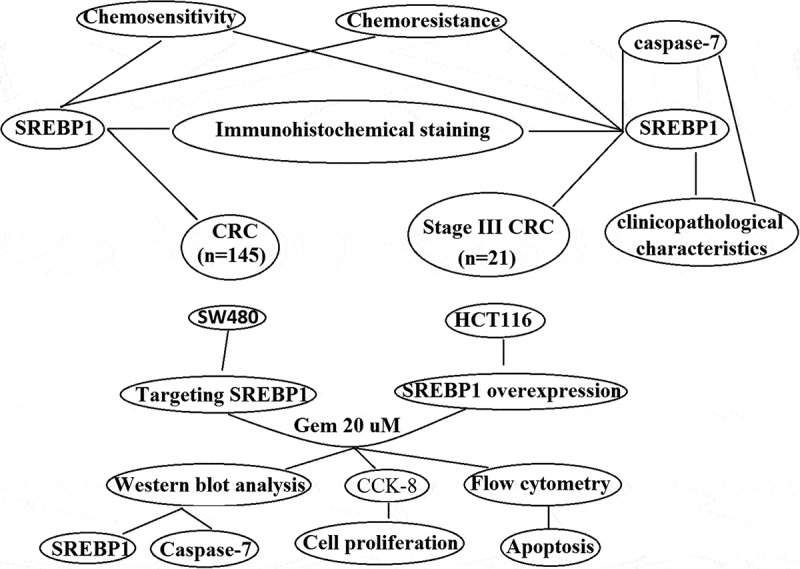

## Introduction

Colorectal cancer (CRC) is one of the most frequent malignancies worldwide, being second in males and third in females in frequency, and ranking fourth and third for cancer-related deaths among males and females, respectively [1]. The age-standardized incidence rate (ASRi) of CRC is higher in men (20.6 per 100,000 individuals) than in women (14.3 per 100,000). The majority of the patients with sporadic malignancies are >50 years of age;75% patients with rectal tumors and 80% patients with colon cancer being ≥60 years of age at the time of diagnosis. The environmental and genetic factors that cause CRC do so by promoting the acquisition of hallmark behaviors of cancer in colonic epithelial cells [2].New treatments for primary and metastatic CRC have been developed and include laparoscopic surgery for primary disease; resection of metastatic disease affecting, for example, the liver and lungs; radiotherapy for rectal cancer and some forms of metastatic disease; and neoadjuvant and palliative chemotherapy [3]. Despite advances in surgical and medical therapies, cure rates and long-term survival have changed little over the past several decades. Against this background, and given that CRC is preceded by a polypoid precursor, screening programs for early detection have gained momentum.

CRC can develop gradually through an accumulation of different somatic or inherited genomic and epigenomic changes. These pathological changes lead to the transformation of colonic mucosa into invasive cancer. There are three highly important molecular pathways leading to CRC development: (1) somatic or germ line derived genomic instability due to inactivation of several tumor suppressor genes such as *APC, SMAD4*, and *TP53*; aberrant DNA methylation, DNA repair defects induced by mutations in mismatch repair genes (MMR); (2) mutational inactivation of tumor suppressor genes (*e.g., APC, TP53, TGFb*, and MMR genes); and (3) over activation of oncogenic pathways including *BRAF, RAS* (*KRAS* and *NRAS*), and phosphatidyl inositol 3-kinase (PIK-3) [4]. The growth and proliferation of metastatic CRC (mCRC) mainly depends on two signaling pathways: the vascular endothelial growth factor (VEGF) and the epidermal growth factor receptor (EGFR) pathways [5].However, the molecular mechanisms by which cancerous development, progression and resistance to chemotherapies occur remain largely unknown.

Sterol regulatory element binding proteins (SREBPs) are a family of transcription factors that regulate lipid homeostasis by controlling the expression of the key and rate-limiting enzymes required for cholesterol and fatty acid (FA) synthesis. In mammals, SREBPs are composed of three isoforms: SREBP1a, SREBP1c, and SRBEP2. SREBP1a and SREBP1c, encoded by a single gene, *SREBF1*, with alternative promoter usage, mainly control lipogenic gene expression, while SREBP2, encoded by a separate gene, *SREBF2*, predominantly regulates cholesterogenic gene expression [6,7]. Lipogenesis is almost universally upregulated in human cancers [8]. Consistent with an essential role of SREBP1 in sensing and regulating intracellular lipid homeostasis, increased expression of SREBP1 has been detected in colorectal carcinoma, breast and prostate cancers, and hepatocarcinoma [9–12]. Moreover, elevated expression of SREBP1 is closely correlated with malignant transformation, cancer progression, and metastasis for several cancer types, particularly hormone-responsive tissue-derived cancers, such as breast and prostate cancers [9,11,13,14], whereas suppression of SREBP1 may inhibit tumor growth [15]. Zhou et al. reported that inhibition of SREBP1 clearly restrains the proliferation of pancreatic cancer (PC) cells, and enhances apoptosis induced by Gemcitabine (Gem). Moreover, SREBP1 suppresses the Gem-induced stemness of PC both *in vitro* and *in vivo*, indicating that inhibition of SREBP1 can be exploited as a novel target for chemoresistance correlated with CSCs in PC [16].

Although Gem has shown preclinical and clinical activity in patients with mCRC, clinical data remain inconclusive. Gem has recognized broad-spectrum activity, and is recommended for treatment of an increasing number of tumor types. However, there is still little clinical evidence of its efficacy in mCRC [17], because of Gem resistance. However, the definitive mechanisms involved in chemotherapy resistance is unknown. In PC cells, Gem treatment induces cell apoptosis by activation of caspase-3/7 signaling, and use of a selective allosteric MEK1/2 inhibitor can enhance Gem efficacy by significant activation of caspase-3/7 [18].In bladder cancer, LNCaP and C4-2B cells, Gem plus AZD7762 also induced more pronounced levels of apoptosis, as indicated by an increase in the levels of cleaved PARP, and of levels of caspase 3/7 activity, compared to Gem alone [19]. Li et al. has recently found that fatostatin induces apoptotic cell death in prostate cancer cells involving SREBP1-dependent caspase-3/7 enzymatic activity [15], suggesting that SREBP1 might regulate caspase 3/7 activity and cell apoptosis in bladder cancer cells. It is not clear whether SREBP1 and caspase-7 have a link involved in chemoresistance in CRC.

In this study, we found that SREBP1 was overexpressed in chemoresistant CRC patient samples. We showed that SREBP1 negatively regulated caspase-7 and sensitized CRC cells to chemotherapy. Low SREBP1 expression was correlated with caspase-7 overexpression in CRC patient samples. Our studies provide important insight into signaling deregulation of the SREBP1- caspase-7 axis in the chemosensitivity of colon cancer, and also help elucidate the role of SREBP1as a therapeutic intervention target in CRC treatment.

### Materials and methods

#### Tissues and patients

21 patients with stage III primary rectal cancer who consecutively underwent neoadjuvant chemotherapy (fluorouracil-leucovorin-oxaliplatin) were randomly selected from the Biobank of the Department of Anorectal, Qilu Hospital of Shandong University. Tumor specimens were obtained by colonoscopy prior to neoadjuvant therapy. The effect of chemotherapy on tumors was assessed as the three-dimensional volume reduction rate or tumor response rate. The tumor response was evaluated by the Response Evaluation Criteria in Solid Tumors (RECIST), which is defined as the following: complete response (CR; disappearance of the disease), partial response (PR; reduction of ≥30%), stable disease (SD; reduction < 30% or enlargement ≤20%), or progressive disease (PD; enlargement ≥20%). Among these, 12 patients were defined as CR/PR and 9 were defined as SD/PD. Paraffin-embedded samples of primary colorectal adenocarcinomas were included involving 145 patients from the Qilu Hospital of Shandong University. Our study protocol was approved by the appropriate ethics committee. Overall survival was the end-point of this study. Survival time was calculated from the date of surgery to the date of death or the last follow-up time. Written informed consent from all patients regarding tissue sampling had been obtained.

#### Immunohistochemistry and evaluation

Paraffin-embedded sections of normal and tumor tissues were deparaffinized in xylene and rehydratedin a decreasing ethanol series diluted in distilled water. Following antigen retrieval with 10 mM citrate buffer, CRC and normal tissue sections were incubated overnight at 4°C with primary anti-SREBP1 or anti-caspase-7 antibodies [Abcam, Cambridge, MA, USA). Following 30 min of incubation with secondary antibody against HRP-conjugated-rabbit Ig, sections were developed in 3, 3ʹ-diaminobenzidinesolution under microscopic observation and counterstained with hematoxylin. The sections were observed under a light microscope for histological review in order to examine tumor microheterogeneity in terms of antigen distribution. Five randomized microscopic views of 400-fold magnification of each section were observed and scored. Both the intensity of immunohistochemical staining (0, negative; 1, weak; 2, intermediate; 3, strong) and the percentage of positive cells (0, 0% positive cells; 1, 1-10% positive cells; 2, 11-50% positive cells; 3, >50%positive cells) were evaluated. The final score for each sample was obtained by multiplying the score of staining intensity and percentage of positive cells. Samples were classified as negative when the final scores were 0 to 3 and positive when 4 to 9. SREBP1 and caspase-7 staining was scored independently by two pathologists blinded to the clinical characteristics of the patients.

#### Cell culture

HCT116 and SW480 cell lines were purchased from the American Type Culture Collection, and grown in RPMI or L15 medium supplemented with 10% fetal bovine serum in a 37°C incubator with a humidified, 5% CO_2_ atmosphere.

#### Lentiviral vector construction

The full length *SREBP1* sequence was synthesized by Shanghai GenePharma Co. and cloned into the pLVX vector (pLVX-SREBP1). Four short hairpin RNA (shRNA) target sequences for *SREBP1* were synthesized by Shanghai GenePharma Co, Ltd and cloned into pLVX vector (pLVX-SREBP1 shRNA). The lentiviral vectors were generated by transient transfection of HEK293T cells with the pLVX-SREBP1, pLVX-SREBP1 shRNAs, and scrambledpLVX packaging plasmids and the relevant transfer plasmid. The harvested HEK293T cell medium was centrifuged at 690 × *g* for 10 min at room temperature and then filtered through a 0.22 µm filter (Nalgene, USA) to remove cell debris. The filtered medium was then harvested and transferred to high speed polyallomer centrifuge tubes (Beckman, USA) and centrifuged at 50,000 × *g* in a SW32Ti rotor (Beckman) for 2 h at 4°C. The vector was then resuspended in DMEM, centrifuged at 1400 × *g* for 10 min and incubated with 5 U/ml DNaseI (Promega, USA) and 10 mM MgCl_2_ (Sigma, UK) for 30 minutes. The vector was then aliquoted and stored at −80°C. The lentiviral titer was determined by serial dilution and transduction of HeLa cells followed by flow cytometry. The packaged lentiviruses were termed Lv- SREBP1 shRNA, Lv- SREBP1, and Lv-scramble. Prior to use, all of the lentiviral vectors were titer matched to 1 × 10^8^ transducing units/ml.

#### Lentivirus infection and siRNA transfection

SW480 cells (high SREBP1 expression) were transfected with Lv- SREBP1 shRNA and Lv-scramble, HCT116 cells (low SREBP1 expression) were transfected with Lv- SREBP1 and Lv-scramble containing 60 MOI for 48 h using Lipofectamine2000following the manufacturer’s instructions. To establish stable transfectants, the cells were exposed to 2 μg/ml puromycin (Sigma, St Louis, MO, USA) for 2 weeks.

To detect the role of SREBP1 on caspase-7 in Lv- SREBP1 shRNA transfected SW480 cells, stably transfected Lv- SREBP1 shRNA or Lv-scramble SW480 cells were transfected with caspase-7 siRNA or control siRNA for 48 h using Lipofectamine 2000 following the manufacturer’s instructions.

#### Drug exposure

Cells were treated with drug vehicle (1% DMSO) or 20 uM Gem for 72 h, or transfected with the Lv- SREBP1, Lv- SREBP1 shRNA, or Lv-scramblefor 24 h before exposure to the same concentration of Gem for 72 h. The concentration and duration of Gem treatment were chosen based on preliminary studies examining its effects on induction of cell apoptosis.

#### Western blot analysis

Cells were lysed and centrifuged. For western blotting, the samples were separated by sodium dodecyl sulfate-polyacrylamide gel electrophoresis (SDS-PAGE), transferred to Immobilon P membranes, and probed with specific antibodies: caspase-7 (D2Q3L) rabbit mAb SREBP1 antibody [Abcam (Cambridge, MA, USA)] and β-actin (Genscript, JiangSu, China). Blots presented here are representative of at least three experiments.

#### Cell proliferation assay

Briefly, 5 × 10^3^ cells per well were plated into 96-well plates and incubated at 37°C, 5% CO_2_ overnight. After transfection, cells were treated with different concentrations of Gem (Sigma) for 48 h. Cell proliferation was measured using the Cell Counting Kit-8 (CCK-8) (Dojindo Co., Kumamoto, Japan) according to the instructions of the supplier. Cells were incubated with CCK-8 for 1 husing 3 replicates, and proliferation rates were assessed by measuring the absorbance at450 nm with a Universal Micro-plate Reader. Each experiment was repeated three times.

#### Flow cytometry

Cells were seeded in 96-well plates at a density of 10^5^ cells per well and cultured overnight. After transfection, cells were treated with different concentrations of Gem for 72 h. Then, the cells were collected and washed twice with PBS by centrifugation at 1000 rpm for 10 min. Cell pellets were resuspended in a FITC-labeled annexin V and propidium iodide (PI) staining solution (BD Bioscience, USA) and incubated for 15 min at room temperature. The samples were then analyzed on a FACSCalibur instrument (FACSCanton II, BD, USA).

#### Statistical analysis

Three independent experiments were performed prior to statistical analysis. The data represent the means ± S.D. *P* < 0.05, by unpaired Student’s *t*-test, was considered statistically significant.

## Results

### SREBP1 expression is correlated with chemoresistance and poor outcome in CRC patients

**Figure 1.**
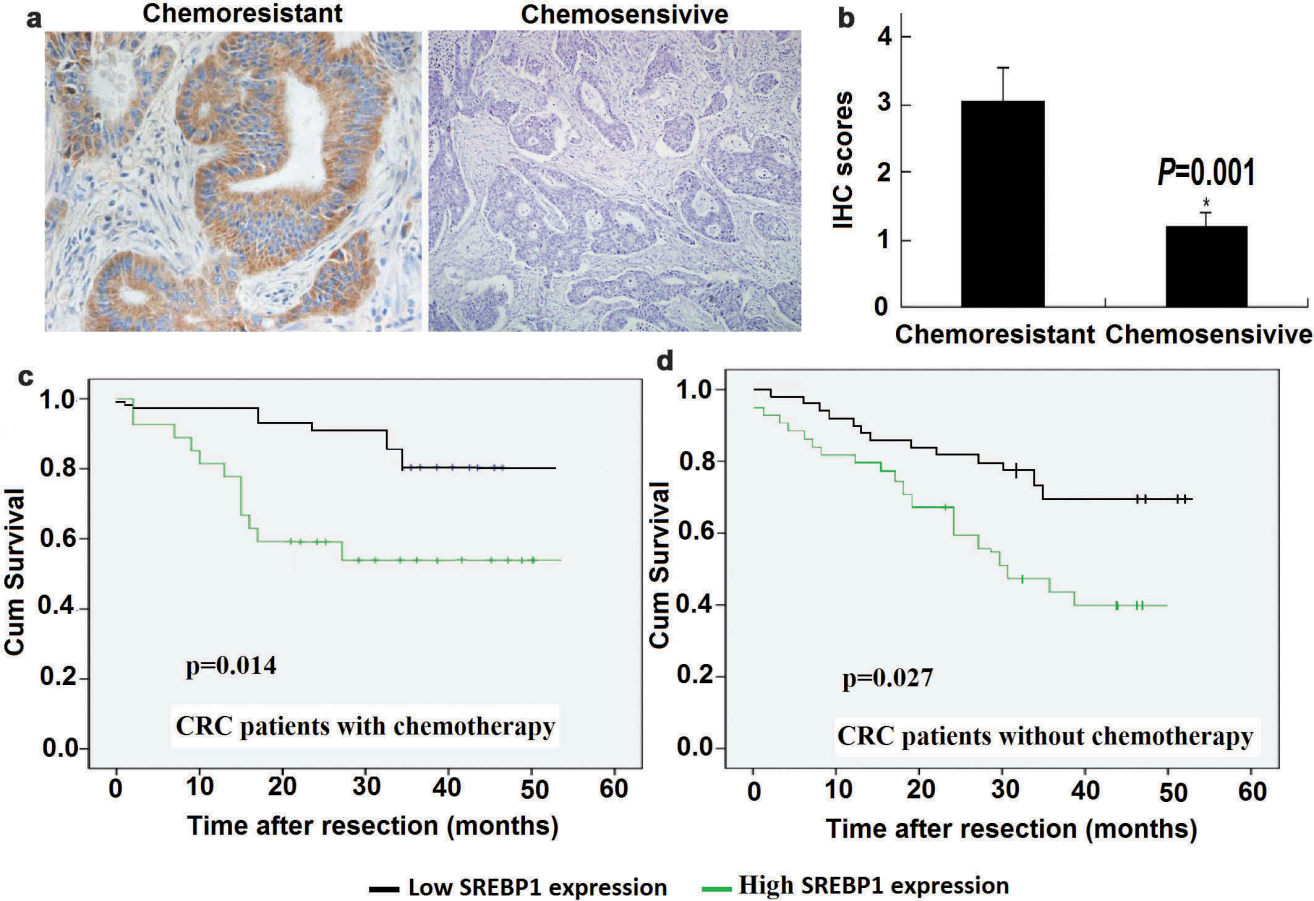
**Chemoresistant colorectal cancer samples express high levels of SREBP1**. a, Representative image of immunohistochemical staining for SREBP1 in chemosensitive and chemoresistant colorectal cancer (CRC) samples. Scale bars: 100μm. b, Statistical analysis revealed IHC scores between the two groups were significantly different (*P* = 0.001). c, d, Kaplan-Meier survival curves of overall survival duration based on SREBP1 expression in CRC tissues. The receiver operating characteristic curve was used to define the cutoff, and log-rank analysis was used to test for significance.

To investigate whether SREBP1 expression relates to chemosensitivity in CRC patients, we first compared the expression levels of SREBP1 in CRC colonoscopy samples from 21 rectal cancer patients who subsequently underwent neoadjuvant chemotherapy. IHC revealed that the level of SREBP1 expression was higher in cancer tissue samples from stable/progressive patients than in samples from complete/partial response patients (Figure 1(a)). Statistical analysis revealed that IHC scores between these two groups were significantly different (Figure 1(b), *P* = 0.001). To determine potential clinical relevance, we performed IHC staining for SREBP1 in 145CRC samples. The clinicopathological characteristics of the CRC patients are summarized in Table 1. Among the CRC patients, 6 of 18 m CRC patients (33.3%) received oxaliplatin based chemotherapy, and 40 of 127 patients (31.5%) without metastasis received chemotherapy. To assess statistical significance, ROC curves were plotted to determine cutoff scores for SREBP1 expression. We divided the CRC patients into high and low SREBP1 expression groups, according to cutoff scores. Among the cohort, high expression of SREBP1 was found in 67 of 145 (46.2%) of CRC patients. There was no significant association between SREBP1 expression and clinicopathological features such as patient gender, age, T classification, N classification, distant metastasis, and clinical stage (Table 1). Kaplan-Meier analysis showed that high expression of SREBP1 was correlated with poor survival, especially in CRC patients who received adjuvant chemotherapy (Figure 1(c,d)). Therefore, SREBP1 can be identified as a biomarker that can predict outcomes in CRC patients.

**Table 1.** Correlation between expression of SREBP1 and clinicopathological features in 145 case of colorectal cancer.

		SREBP1 expression		
	All cases	Low(n)	High(n)	p-value
**Gender** Male	76	40	36	0.23
Female	69	38	31	
**Age(Year)**				
<60	63	36	27	0.34
≥60	82	42	40	
**Histological grade**				
G1	14	8	6	0.46
G2	105	54	51	
G3	26	16	10	
**pT status**				
T1-T2	28	14	14	0.11
T3-T4	117	64	53	
**pN status**				
N0	86	48	38	0.17
N1	59	30	29	
**pM status**				
M0	127	67	60	0.13
M1	18	11	7	
**Clinical stage**				
I/II	80	40	40	0.086
III/IV	65	38	27	
**Chemotherapy**				
No	99	55	44	0.23
Yes	46	23	23

### SREBP1 levels are increased in HCT116 cells in response to Gem

**Figure 2.**
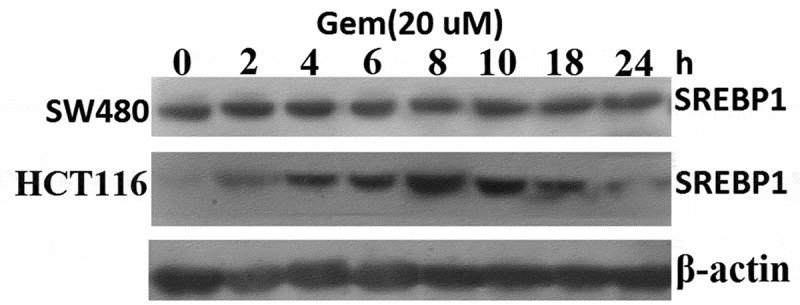
**Gem-induced SREBP1 expression in HCT116 and SW480 cells**.HCT116 and SW480 cells were treated with 20 uM Gem for 2–24 h, SREBP1 expression was detected by western blot assay.

In order to understand how SREBP1 levels are regulatedin CRC, we chose to study SREBP1regulation in the colon cancer cell lines HCT116 and SW480 underconditions where cells are subjected to stress induced by Gem. Treatment ofHCT116 cells (lowendogenous SREBP1 expression) with 20 uM Gem for 24 h did indeed result in a significant increase in endogenous SREBP1 protein levels (Figure 2), with the increase occurring after 2 h treatment of HCT116 cells with Gem, and attained the highest level at 8 h, and then gradually decreased and reached the lowest level by 24 h. A less pronounced effect occurred with Gem in SW480 cells (high endogenous SREBP1 expression) (Figure 2).

### SREBP1 knockdown leads to chemosensitivity of SW480 cells

**Figure 3.**
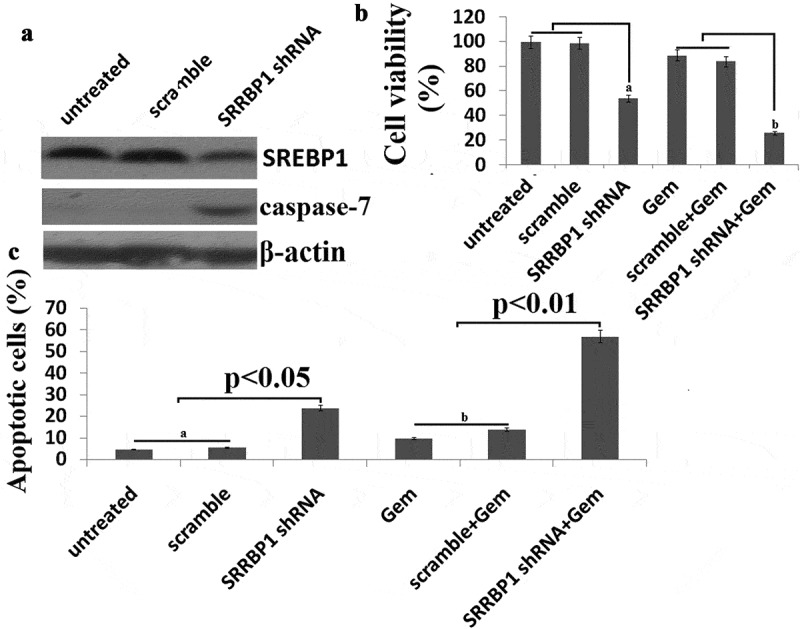
**SREBP1 knockdown increases chemosensitivity of SW480 cells to Gem**. a, SW480 cells were transfected into Lv- SREBP1 shRNA or Lv-scramble for 48 h, SREBP1 and caspase-7 was detected by western blot assay. b, SW480 cells were transfected into Lv- SREBP1 shRNA or Lv-scramble for 24 h, then treated with 20 uM Gem for 72 h. Cell proliferation assay of SW480 cells using cell counting kit-8 assay. c, SW480 cells were transfected into Lv- SREBP1 shRNA or Lv-scramble for 24 h, then treated with 20 uM Gem for 72 h. Cell apoptosis was analyzed on a FACS Calibur. Data are shown as mean±SD.

To examine further whether SREBP1 expression has an impact on chemoresistance of colon cancer cells *in vitro*, SREBP1 was downregulated in SW480 cells. We found that SW480 cells were resistant to Gem treatment. SW480 cells were transfected with Lv-SREBP1 shRNA or Lv-scramble for 24 h (Figure 3(a)). Then, the cells were treated with 20 uM Gem for 72 h. Chemosensitivity was assayed using Cell Counting Kit-8.Results showed that knockdown of SREBP1 significantly increased chemosensitivity of SW480cells to Gem (Figure 3(b)).Apoptosis of SW480 cells treated with 20 μg Gem for 72h was assayed by PI staining and flow cytometry (Figure 3(c)).Cellular apoptosis was significantly increased in Lv-SREBP1 shRNA transfected SW480 cells. This indicated that knockdown of SREBP1significantly increased Gem-induced apoptosis. Knockdown of SREBP1 also decreased cell growth and apoptosis *in vitro* (Figure 3(a,b)).

### SREBP1 overexpression leads to chemoresistance of HCT116 cells

**Figure 4.**
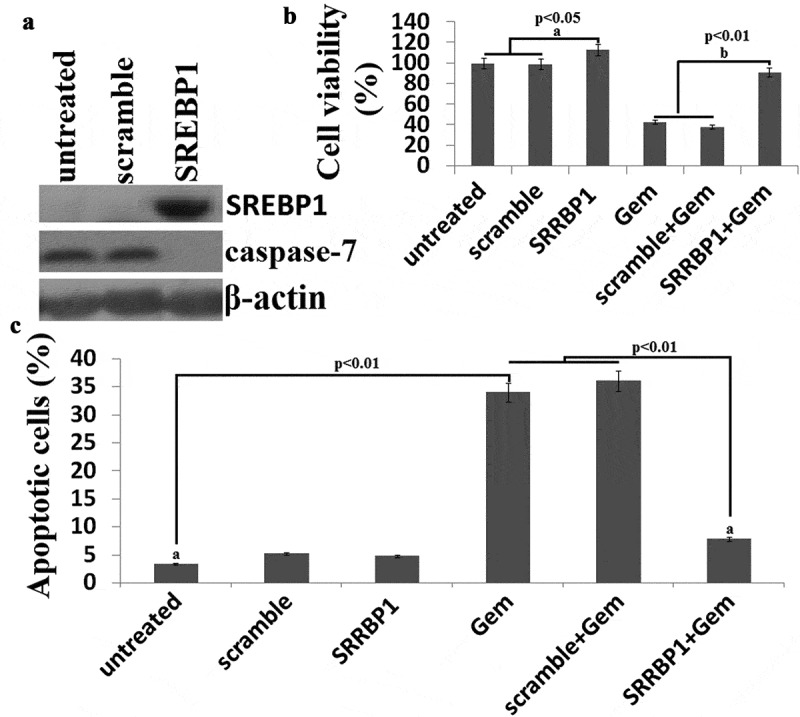
**SREBP1 overexpressionincreases chemoresistance of HCT116 cells to Gem**. a, HCT116 cells were transfected into Lv- SREBP1 or Lv-scramble for 48 h, SREBP1 and caspase-7 was detected by western blot assay. b, HCT116 cells were transfected into Lv- SREBP1 or Lv-scramble for 24 h, then treated with 20 uM Gem for 72 h. Cell proliferation assay ofHCT116 cells using cell counting kit-8 assay. c, HCT116 cells were transfected into Lv- SREBP1 or Lv-scramble for 24 h, then treated with 20 uM Gem for 72 h. Cell apoptosis was analyzed on a FACSCalibur. Data are shown as mean±SD.

We examined whether SREBP1overexpression had an impact on the chemoresistance of colon cancer cells. We found thatHCT116 cells with lower endogenous SREBP1 expression were sensitive to Gem treatment.HCT116 cells were transfected with Lv-SREBP1 or Lv-scramble for 24 h(Figure 4(a)). Then, the cells were treated with gradient concentrations of Gem for72 h and the chemosensitivity was assayed. Results showed thatSREBP1overexpression promoted cell viability and significantly decreased chemosensitivity of HCT116cells to Gem (Figure 4(b)). Apoptosis of HCT116 cells treated with 20 μg Gem for 72hours was assayed by PI staining and flow cytometry. Cell apoptosis was restored in the Lv-SREBP1-transfected HCT116 cells (Figure 4(c)). This indicated that SREBP1 overexpression significantly decreased Gem-induced apoptosis. Taken together, these results indicated that SREBP1 expression leads to chemoresistance.

### SREBP1 negatively regulates caspase-7

**Figure 5.**
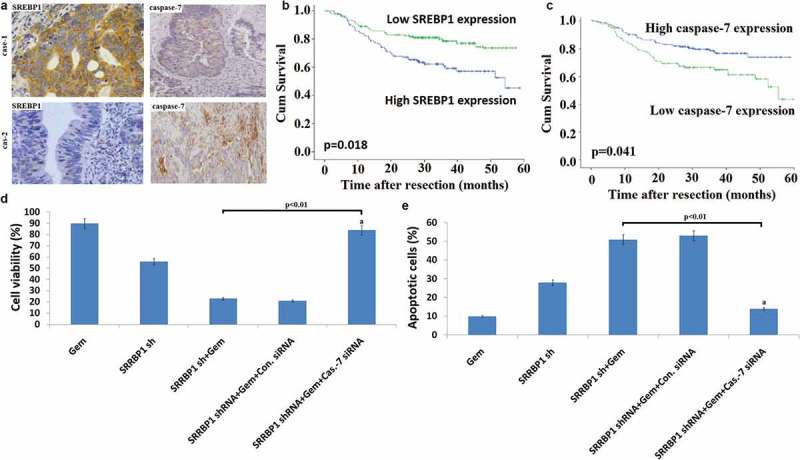
**SREBP1 regulates caspase-7 expression and colon cancer chemoresistance**. a, Representative IHC staining for SREBP1 and caspase-7 in serial sections from colon cancer patient samples. The sample used was derived from colon cancer cases. Scale bars represent 50μm. b, Kaplan-Meier survival curves of overall survival duration based on SREBP1 expression in the CRC patient tissues. c, Kaplan-Meier survival curves of overall survival duration based oncaspase-7 expression in the CRC patient tissues.The receiver operatingcharacteristic curve was used to define the cutoff, and log-rank analysis was used to test for significance. d, SW480 cells were co-transfected into Lv- SREBP1 shRNA and caspase-7 siRNA for 24 h, then treated with 20 uM Gem for 72 h. Cell proliferation assay of SW480 cells using cell counting kit-8 assay. e, SW480 cells were co-transfected into Lv- SREBP1 shRNA and caspase-7 siRNA for 24 h, then treated with 20 uM Gem for 72 h. Cell apoptosis was analyzed on a FACSCalibur. Data are shown as mean±SD.

To determine clinical relevance, we subjected 145 human CRC specimens to IHC staining for caspase-7 and SREBP1. The CRC samples had high SREBP1 expression, which correlated with low caspase-7 expression (representative case 1). Accordingly, CRC samples with low SREBP1 expression had high caspase-7expression (representative case 2) (Figure 5(a)). SREBP1 and caspase-7 expression levels were inversely correlated with each other (Table 2). Importantly, Kaplan-Meier analysis showed that high expression of SREBP1 and low expression of caspase-7 were correlated with poor survival (Figure 5(b,c)). These results strongly suggested that the SREBP1-caspase-7 axis is deregulated during the development of human CRC.

**Table 2.** Correlation between SREBP1 and caspase-7 expression in CRC patients samples.

	Low caspase-7	High caspase-7	Total	P value
Low SREBP1 High SREBP1	60 26	18 41	78 67	
Total	86	59	145	0.019

Because caspase-7 can regulate the chemosensitivity of cancer cells, we explored whether caspase-7 is involved in SREBP1-mediated chemosensitivity. Caspase-7 levels were decreased when HCT116 cells were transfected with Lv-SREBP1 (Figure 3(a)). Furthermore, caspase-7 levels were increased when SW480 cells were transfected with Lv-SREBP1 shRNA (Figure 4(a)).

To define whether caspase-7 was required for SREBP1-mediateddrug resistance in CRC, we performed a rescue experiment by knocking down caspase-7 by siRNA in Lv-SREBP1shRNA transfected SW480 cells followed by testing for cellular proliferation and apoptosis. The results of this rescue experiment demonstrated that blockade ofcaspase-7 significantly diminished the effect of SREBP1 downregulation on Gem-induced SW480cell proliferation and apoptosis (Figure 5(d,e)).

## Discussion

Adjuvant chemotherapy with 5FU and oxaliplatin currently remains a standard treatment for patients with advanced CRC. However, chemotherapy resistance leading to treatment failure and local recurrence is still a critical problem. One of the biggest challenges is to identify the subpopulation of patients who are most likely to respond to a specific therapy. If one or more biomarkers could predict patient response to chemotherapy, one could spare the non-responders from ineffective treatment and direct them to alternative treatment strategies that could be more effective.

The original cloning of the *SREBP1* gene from human HeLa cells yielded several partial cDNA clones with alternative sequences at both the 5ʹ and 3ʹ ends, which were postulated to result from alternative splicing.SREBP1 consists of SREBP1a and SREBP1c, which are encoded by the same gene (*SREBP1*), regulated by two distinct promoters and alternative splicing [20].The two different sequences at each end were designated ‘a’ and ‘c’. The full-length human cDNA containing ‘c’ sequences at both ends was designated *SREBP1c*, and the full-length cDNA isolated from Chinese hamster ovary (CHO) cells containing ‘a’ sequences at both ends was designated SREBP1a [21]. The *N*-terminus contains a DNA activation domain which can bind transcriptional coactivators, such as CBP [22]. SREBP1a activates genes required in the synthesis of cholesterol and fatty acid, such as elongase and stearoyl-CoA desaturase, while SREBP1c directly activates the expression of more than 30 genes dedicated to the synthesis and uptake of FAs and triglycerides.SREBP-1a is expressed at a higher level in comparison to SREBP-1c in proliferating cells, such as cancer cells, spleen, and intestinal tissues [23] and is implicated as a regulator of phospholipid biogenesis genes in mammals [24], whereas SREBP-1c is the predominant form of the *SREBF1* gene product *in vivo*, particularly in hepatocytes.

SREBP1 has been shown to be involved in tumorigenesis. Increased expression of SREBP1 has been reported in colorectal carcinoma, breast and prostate cancer and hepatocarcinoma. The levels of SREBP1 positively correlate with the severity of endometrial hyperplasia and cancer, which further supports the notion that SREBP1 is oncogenic. Despite this progress, the role of SREBP1 in endometrial tumorigenesis remains unexplored. There is some evidence that SREBP1 can modulate drug metabolism and thus influence anti-tumor therapy [25–27]. Cancer cell responses to chemotherapy are closely correlated to the functional status of the *SREBP1* gene [25,26].

In the present study, we found that SREBP1 is overexpressed in chemoresistant CRC. Furthermore, high expression of SREBP1 was significantly associated with poor chemotherapeutic outcomes in CRC patients who received standard adjuvant chemotherapy. However, targeting SREBP1 sensitized CRC cells to Gem and *vice versa*, demonstrating thatSREBP1 may play an important role in CRC chemoresistance.

SREBF1 was found to serve as a regulatory hub that controls lipid metabolism, cell growth, and cell death [28]. *Caspase-7* was found to be an SREBF-responsive gene, raising the possibility for caspase cascade to participate in the control of cholesterol/triacylglycerol levels [29]. Activated caspases lead to induction of early stage apoptotic cascade, suggesting that these proteins may play a role in the proper execution of the apoptotic program [30].

In our study, we found that SREBP1 overexpression is quite widespread in chemoresistant CRC. SREBP1 overexpression was negatively correlated with caspase-7 protein expression in human colon cancer samples. These findings demonstrated that caspase-7 downregulation can at least partially account for SREBP1 overexpression in colon cancer, which provides important insights into the mechanisms underlying SREBP1 overexpression in chemoresistant CRC. The inverse relationship between SREBP1 and caspase-7 in CRC was demonstrated for the first time in our study. Our *in vitro* study showed that targeting SREBP1 promoted caspase-7 expression in SW480 cells with high endogenous SREBP1 expression, and SREBP1 overexpression inhibited caspase-7 expression in HCT116 cells with low endogenous SREBP1 expression. Furthermore, targeting caspase-7 in SW480 cells led to increased cell viability and decreased apoptosis in the presence of SREBP1 downregulation, thereby resulting in decreased sensitivity to chemotherapy drugs, suggesting that targeting SREBP1 sensitizes CRC cells to chemotherapy by caspase-7 upregulation.

### Conclusion

We found in our study that SREBP1 overexpression is associated with poor prognosis and chemoresistance in patients with CRC. TargetingSREBP1 is an efficient therapeutic approach to overcoming chemoresistance.

## Highlights

- SREBP1 overexpression is correlated with chemoresistance and poor prognosis in CRC patients
- Low caspase-7 expression is correlated with poor patient survival
- TargetingSREBP1 increases chemosensitivity to Gem by upregulationof caspase-7in CRC cells
- SREBP1 could be a biomarker for predicting chemotherapy response
